# HDAC Inhibition Regulates Cardiac Function by Increasing Myofilament Calcium Sensitivity and Decreasing Diastolic Tension

**DOI:** 10.3390/pharmaceutics14071509

**Published:** 2022-07-21

**Authors:** Deborah M. Eaton, Thomas G. Martin, Michael Kasa, Natasa Djalinac, Senka Ljubojevic-Holzer, Dirk Von Lewinski, Maria Pöttler, Theerachat Kampaengsri, Andreas Krumphuber, Katharina Scharer, Heinrich Maechler, Andreas Zirlik, Timothy A. McKinsey, Jonathan A. Kirk, Steven R. Houser, Peter P. Rainer, Markus Wallner

**Affiliations:** 1 Cardiovascular Research Center, Lewis Katz School of Medicine, Temple University, Philadelphia, PA 19140, USA; deborah.eaton@pennmedicine.upenn.edu (D.M.E.); srhouser@temple.edu (S.R.H.); 2 Penn Cardiovascular Institute, University of Pennsylvania Perelman School of Medicine, Philadelphia, PA 19104, USA; 3 Department of Cell and Molecular Physiology, Loyola University Chicago Stritch School of Medicine, Chicago, IL 60153, USA; thomas.martin-2@colorado.edu (T.G.M.); june.thk39@gmail.com (T.K.); jkirk2@luc.edu (J.A.K.); 4 Division of Cardiology, Medical University of Graz, 8036 Graz, Austria; michael.kasa@gmx.at (M.K.); natasa.djalinac@medunigraz.at (N.D.); senka.ljubojevic@medunigraz.at (S.L.-H.); dirk.von-lewinski@medunigraz.at (D.V.L.); maria.poettler@medunigraz.at (M.P.); andreas.krumphuber@stud.medunigraz.at (A.K.); katharina.scharer@stud.medunigraz.at (K.S.); andreas.zirlik@medunigraz.at (A.Z.); peter.rainer@medunigraz.at (P.P.R.); 5 Department of Cardiothoracic Surgery, Medical University of Graz, 8036 Graz, Austria; heinrich.maechler@medunigraz.at; 6 Division of Cardiology, Department of Medicine, University of Colorado Anschutz Medical Campus, Aurora, CO 80045, USA; timothy.mckinsey@cuanschutz.edu; 7 Consortium for Fibrosis Research & Translation, University of Colorado Anschutz Medical Campus, Aurora, CO 80045, USA; 8 BioTechMed Graz, 8010 Graz, Austria

**Keywords:** heart failure, contractility, calcium, cardiomyocyte, myofilament, HDAC inhibitor

## Abstract

We recently established a large animal model that recapitulates key clinical features of heart failure with preserved ejection fraction (HFpEF) and tested the effects of the pan-HDAC inhibitor suberoylanilide hydroxamic acid (SAHA). SAHA reversed and prevented the development of cardiopulmonary impairment. This study evaluated the effects of SAHA at the level of cardiomyocyte and contractile protein function to understand how it modulates cardiac function. Both isolated adult feline ventricular cardiomyocytes (AFVM) and left ventricle (LV) trabeculae isolated from non-failing donors were treated with SAHA or vehicle before recording functional data. Skinned myocytes were isolated from AFVM and human trabeculae to assess myofilament function. SAHA-treated AFVM had increased contractility and improved relaxation kinetics but no difference in peak calcium transients, with increased calcium sensitivity and decreased passive stiffness of myofilaments. Mass spectrometry analysis revealed increased acetylation of the myosin regulatory light chain with SAHA treatment. SAHA-treated human trabeculae had decreased diastolic tension and increased developed force. Myofilaments isolated from human trabeculae had increased calcium sensitivity and decreased passive stiffness. These findings suggest that SAHA has an important role in the direct control of cardiac function at the level of the cardiomyocyte and myofilament by increasing myofilament calcium sensitivity and reducing diastolic tension.

## 1. Introduction

Heart failure (HF) represents a major global health crisis. There are currently an estimated 26 million adults living with heart failure worldwide and this number is predicted to rise in the future [1]. About half of all heart failure patients are diagnosed with heart failure with preserved ejection fraction (HFpEF) and the other half with heart failure with (mildly) reduced ejection fraction (HFmrEF, HFrEF) [2]. There is a stark contrast in the etiology of the syndrome and treatment options available for patients with HFrEF and HFpEF. While there are several drug classes that have proven to effectively improve mortality in HFrEF, until recently no therapeutics have improved the outcome in patients with HFpEF. EMPEROR-Preserved, a randomized clinical trial comparing empagliflozin vs. placebo in HF patients with an EF greater or equal to 40%, produced a positive outcome [3]. To address this lack of treatments, we must bridge the gap from basic science where novel therapeutic targets are identified to pre-clinical and translational medicine. Part of the solution involves developing representative animal models that capture multiple aspects of the complex human syndrome of HFpEF [4]. Our lab previously published an in-depth characterization of the feline model of slow progressive pressure overload, which recapitulates several key clinical features of HFpEF [5,6]. While these animals have preserved ejection fraction, they develop systolic and diastolic dysfunction when assessed using speckle-tracking-based strain analysis and invasive hemodynamics. Aortic banded animals also develop pulmonary hypertension secondary to elevated LV filling pressures, which is accompanied by structural remodeling, reduced lung compliance, increased intrapulmonary shunting, and impaired oxygenation. This robust and extensively characterized cardiopulmonary phenotype is a useful platform for testing potential therapies for heart failure [6].

While developing new treatments is important, repurposing Federal Drug Administration (FDA)/European Medicine Agency (EMA)-approved therapies for HF has gained attention in recent years as these drugs have already been clinically evaluated for other diseases [7]. In line with this idea, we decided to focus on epigenetic/post-translational modifications as a therapeutic target by testing suberoylanilide hydroxamic acid (SAHA, vorinostat), which is a pan-histone deacetylase (HDAC) inhibitor, and was the first in class therapy to earn FDA approval in 2006 as a treatment for cutaneous T-cell lymphoma [8]. HDACs are enzymes that have genomic and non-genomic effects. Deacetylation of histones by HDACs leads to chromatin compaction, acting as an off switch for mRNA synthesis and regulating gene expression. HDACs can also facilitate post-translational protein modifications by catalyzing the removal of lysine acetyl groups from ε-amino groups of lysine residues [9,10]. HDACs are activated in cardiomyocytes subjected to pressure overload [11]. Using HDAC inhibitors to harness this integral catalytic activity is a promising therapeutic strategy. We previously showed that SAHA treatment improved systolic and diastolic cardiac, pulmonary, and mitochondrial function in an animal model of HFpEF [5]. More in-depth analyses provided a potential mechanism for the improvement in diastolic function. Using isolated myofibrils, we found a significant improvement in the linear relaxation duration with SAHA. Linear relaxation duration represents inactivation of thin filament regulatory proteins after Ca^2+^ unbinds [12]. This finding indicates that SAHA may have a direct role in modulating diastolic function via direct control of relaxation properties of myofibrils.

However, mechanisms and substrates for acetylation/deacetylation responsible for the improvements in systolic and diastolic cardiac function are poorly understood. Several studies have assessed the effect of SAHA in cardiomyocytes isolated from rodents and provided valuable insight [13,14], but there were still many unanswered questions. Furthermore, no study has assessed the functional effects of SAHA on human cardiac tissue. To address this gap in knowledge, we performed an in-depth analysis using tissue and cellular components from both non-failing human hearts and a large mammalian model with comparable physiological properties (action potential duration) and myosin heavy chain composition [15,16]. Our aim was to develop a better understanding of how SAHA treatment modulates systolic and diastolic function at a cellular and molecular level and its contractile components and identify potential substrates for acetylation/deacetylation.

## 2. Materials and Methods

Figure S1 details the experimental design.

### 2.1. AFVM Isolation

AFVMs were isolated from the left ventricle of domestic short hair kittens (n = 4, 6 months old) (Marshall Laboratories) as previously described [5,17,18,19]. Animals were sedated with 50 mg/kg of ketamine and 0.1 mg/kg of acepromazine (intramuscular), then 50 mg/kg of sodium pentobarbital (intraperitoneal). The heart was excised via cardiectomy, washed in ice cold Krebs-Henseleit Buffer (KHB) (12.5 mmol/L glucose, 5.4 mmol/L KCl, 1 mmol/L lactic acid, 1.2 mmol/L MgSO_4_, 130 mmol/L NaCl, 1.2 mmol/L NaH_2_PO_4_, 25 mmol/L NaHCO_3_, and 2 mmol/L Na-pyruvate, pH 7.4) and then the aorta was cannulated. The coronary arteries were then flushed with KHB to clear out any remaining blood. The heart was retrograde perfused with KHB using a modified Langendorff apparatus, then switched to digestion buffer (KHB, 180 units/mL collagenase type II (Worthington Biochemical Corporation, NJ, USA) and 50 µmol/L CaCl_2_). All solutions were heated to 37 °C prior to perfusion through the heart and aerated with 95% oxygen and 5% CO_2_ to maintain pH at 7.4. The heart was continuously checked to monitor digestion and then the left ventricle was separated from the rest of the heart and minced. The cardiomyocytes were then filtered, resuspended, and equilibrated in room temperature KHB with 200 µmol/L CaCl_2_, and 1% bovine serum albumin (BSA) added.

### 2.2. AFVM Acute SAHA Treatment and Contractility/Calcium Recordings

Once AFVM had settled via gravity, they were washed with Normal Tyrode’s solution (NT) (10 mM glucose, 5 mM HEPES, 5.4 mM KCl, 1.2 mM (MgCl_2_) (6H_2_O), 150 mM NaCl, and 2 mM NA-pyruvate, pH 7.4) with 1mM CaCl_2_ (NT + 1 mM CaCl_2_) added. A stock solution of 5 mM SAHA was prepared and then serially diluted to 2.5 µM of SAHA using NT + 1 mM CaCl_2_. Corresponding dilutions of DMSO were prepared in NT + 1 mM CaCl_2_ to use as the vehicle treatment. Cells were treated with SAHA or vehicle and incubated for 90 min. After 90 min, cells were incubated with Fluo-4am (ThermoFisher Scientific, Waltham, MA, USA) and Pluronic™ F-127 (ThermoFisher Scientific) for 12–15 min. Cells were then loaded onto a heated chamber of an inverted microscope and perfused with NT + 1 mM CaCl_2_ with 2.5 µM SAHA or corresponding DMSO dilution added. Cells were first paced at 0.5 Hz to check for any not following stimulation. Once the steady state was reached, a minimum of 15 contractions was recorded using the IonOptix Calcium and Contractility platform. Background fluorescence was recorded for all cells. Analysis was performed offline using the IonOptix IonWizard software. Background fluorescence was subtracted from the calcium recording prior to performing calculations during analysis. Peak calcium transient was calculated as F/F_0_, where F is peak calcium and F_0_ is baseline calcium. Fractional shortening was calculated as ((baseline sarcomere length—peak sarcomere length/baseline sarcomere length) × 100).

### 2.3. Trabeculae Harvest

Trabeculae were isolated from the ventricles of human non-failing donor hearts that were not suitable for transplantation (n = 7 patients) or right atrial appendages (n = 30) as previously described [20]. A small piece of myocardium (5–8 mm × 5–8 mm) was excised and immediately stored in ice-cold cardioplegic solution for transportation to the lab. Using a stereomicroscope, small endocardial trabeculae (cross-sectional area < 0.8 mm^2^) (LV: n = 18, RA: n = 28) were dissected off the larger piece of tissue.

### 2.4. Trabeculae Developed Force Experiments

The individual trabeculae were then moved to an organ bath where it was mounted on miniature hooks attached to a force-transducer and superfused with modified Tyrode’s solution at 37 °C (127 mM NaCl, 2.3 mM KCl, 25 mM NaHCO_3_, 0.6 mM MgSO_4_, 1.3 mM KH_2_PO_4_, 2.5 mM CaCl_2_, 11.2 mM glucose, 5IE/L insulin). The trabeculae were then electrically stimulated (STM1 Scientific Instruments) at 1 Hz (voltage 25% over threshold) and gradually stretched to reach the optimum preload, which is considered baseline. Developed force (systolic force—diastolic force) was recorded using a force transducer (Scientific instruments) and stored digitally (Labview) as well as with a thermorecorder (Graphtec Linearcorder WR 3320) for offline analysis. Baseline measurements were recorded and then trabeculae were treated with 10 µM SAHA or vehicle (DMSO) for 120 min. For selective HDAC inhibitor studies, data was collected using both low (Rodin-A: 2 µM; IRBM-D: 100 nM) and high (Rodin-A: 10 µM; IRBM-D: 250 nM) concentrations, in addition to a control group (DMSO 10 µM). Measurements were recorded at 15 min intervals for the entire 120 min treatment period for all trabeculae experiments. In profiling against isolated recombinant HDAC isoforms for inhibition of enzymatic activity, Rodin-A selectively inhibited the HDAC1 and HDAC2 isoforms. The half maximum inhibitory concentration (IC50) for HDAC1 and HDAC2 was 0.15 µM and 0.43 µM, respectively. IRBM-D selectively inhibited the HDAC1, HDAC2, and HDAC3 isoforms. The IC50 for HDAC1, HDAC2, and HDAC3 was 13 nM, 18 nM, and 11 nM, respectively. Detailed pharmacokinetic data has been previously reported [21,22].

### 2.5. Skinned Myocyte Functional Experiments

Skinned cardiomyocytes from human trabeculae were prepared as described previously [23]. Briefly, tissue was suspended in Isolation solution (108 mM KCl, 10 mM Imidazole, 8.9 mM KOH, 7.1 mM MgCl_2_, 5.8 mM ATP, 2 mM EGTA) containing 0.5% Triton and 1:100 protease/phosphatase inhibitors (Halt, Thermo Fisher Scientific) and mechanically homogenized at 7000 RPM. The homogenate was then passed through a 70 µm filter and left on ice for 20 min. The filtered myocytes were pelleted by centrifugation at 120 RCF for 2 min and then resuspended in isolation solution without Triton. For the isolated adult feline ventricular myocytes (AFVMs), this same procedure was adopted except for mechanical homogenization and filtering.

To perform the active tension experiments, single cardiomyocytes were attached to two pins (one attached to a calibrated force transducer and the other to a piezo length controller) with UV-curing glue (Thor Labs, Newton, NJ, USA). The myocyte was then perfused with solutions of varying calcium concentration and the force exhibited by the cell in response to each was recorded and normalized to the myocyte cross-sectional area. The data were fit to a Hill equation to determine the F_max_ and EC_50_. All experiments were performed at a sarcomere length of 2.1 µm and at room temperature. For the AFVM, n = 3 felines. For non-failing human, n = 7 patients.

To determine the myocyte passive tension, the cell was moved to a bath free of calcium. Using the length controller pin, the cell was next stretched in 0.2 µm increments from resting sarcomere length of 1.6–2.8 µm. The passive force at each sarcomere length was measured with the force transducer and normalized to the myocyte cross-sectional area. The data were fit to an exponential growth equation. These experiments were performed at room temperature. For the AFVM, n = 3 felines. For non-failing human, n = 7 patients.

### 2.6. Myofilament Protein Enrichment

Tissue was homogenized at 7000 RPM in a standard rigor buffer (SRB) containing 0.5% Triton and 1:100 protease/phosphatase inhibitors and left on ice for 20 min. The homogenate was then centrifuged at 1800 RCF to pellet the myofilament proteins. The pellet was washed twice in triton-free SRB by resuspension and centrifugation at 1800 RCF. The myofilament protein pellet was next solubilized in 9M urea, sonicated, and centrifuged at 10,000 RCF. The now solubilized myofilament proteins were collected as the supernatant and stored in aliquots at −80 °C. Protein concentration was determined by Pierce BCA Assay (Thermo Fisher Scientific).

### 2.7. Myofilament Immunoblots

Samples of 20 µg myofilament protein were combined in a 1:1 (*v*/*v*) ratio with SDS-Tris Glycine gel loading buffer (Novex) supplemented 1:5 with Bolt reducing buffer (Novex) and heated at 95 °C for 10 min. The samples were then loaded on 4–12% Bis Tris Plus gradient gels and proteins were separated by electrophoresis at 200 volts and then transferred onto a nitrocellulose membrane. The membrane was incubated in Revert Total Protein Stain (LICOR) to verify equal loading and serve as a loading control and then blocked for 1 h at room temperature in 1:1 0.1% Tween-TBS/Intercept TBS blocking buffer (LICOR). The Anti-Lysine Acetylation antibody (Cytoskeleton, 19C4B2.1) was added at 1:500 dilution in blocking buffer and incubated overnight at 4 °C. The blot was washed in TBS-T and then IR-dye donkey anti-mouse 680RD secondary antibody (LICOR) was added at 1:10,000 dilution for 1 hour at room temperature. The results were visualized using the Azure c600 imaging system and analyzed using LICOR Image Studio. For analysis, the lysine-acetylation signal was normalized to the total protein signal.

### 2.8. Mass Spectrometry

Myofilament protein lysates were separated by SDS-PAGE as above. The gel was then fixed in 50% methanol/10% acetic acid for 15 min and incubated in Coomassie Brilliant Blue for 30 min with gentle agitation, followed by destaining overnight (10% methanol, 7.5% acetic acid). Approximately 24 h after adding the destain solution, the gel was washed with HPLC-grade water twice for 1 h each.

The bands of interest (20 kDa) were cut from the gel and added to 1.5 mL Eppendorf tubes containing 100 mM ammonium bicarbonate. The tubes were incubated at 37 °C for 15 min and 600 RPM in a shaking incubator. The solution was discarded, replaced with a 1:1 (*v*/*v*) ammonium bicarbonate/acetonitrile solution, and then incubated again in the same fashion. After 15 min, a 100% acetonitrile solution was added, and the shaking incubation repeated. These three incubations constitute the “wash” step, which is repeated later. The gel pieces were next suspended in 250 mM DTT in 100 mM ammonium bicarbonate and incubated at 56 °C for 1 h and 600 RPM to reduce disulfide bonds. Alkylation was performed with 50 mM iodoacetamide in 100 mM ammonium bicarbonate at room temperature for 45 min in a dark room. Following this incubation, the iodoacetamide was removed and the wash step detailed above was repeated.

To digest the protein, 5 µg of Trypsin/LysC protease was added to the gel pieces in 200 µL 100 mM ammonium bicarbonate and incubated at 37 °C for 18 h and 600 RPM. The peptide-containing solution was then collected, and the gel pieces were washed twice in 50% acetonitrile/5% formic acid to collect the remaining peptides. The peptide solutions were dried by vacuum centrifugation, reconstituted in 3% acetonitrile/0.1% formic acid, and ~100 ng was subjected to high pressure liquid chromatography in a 25 cm PepMap RSLC C18 column coupled to tandem mass spectrometry on a LTQ Orbitrap XL mass spectrometer. Three technical replicates were run for each of the three samples in the vehicle and SAHA treatment groups. The acquired raw data files were analyzed using the Peaks Bioinformatics software and matched to the *Felis catus* proteome FASTA database downloaded from Uniprot. The fixed modification was cysteine carbamidomethylation (+57.02) and the variable modification was lysine acetylation (+42.01). For analysis, acetylated RLC peptides were normalized to total RLC peptides identified for each sample. Outliers greater than two standard deviations from the mean were removed.

### 2.9. Western Blot for Calcium Handling Proteins

Right atrium samples (n = 8 patients) were homogenized in homogenization buffer and protein concentration was determined using Pierce BCA assay (Thermo Fisher Scientific). A total amount of 10 µg of protein per sample was loaded on a 4–12%—Bis-TRIS Gradient Gel (BioRad, Hercules, CA, USA) or in case of PLB a 16.5% TRIS-TRICINE-Gel (BioRad). Samples were transferred to nitrocellulose membranes (Amersham Protram) of 0.45 µm or 0.1 µm for PLB and probed with the following antibodies: GAPDH (Cell Signaling Technology #5174, Danvers MA, USA), RyR_2808_Ser (Badrilla #A010-30, Leeds, UK), phCaMKII Thr286 (Abcam #32678, Cambridge, UK), Serca2a (Badrilla #A010-20), Acetylated Lysine (Cell Signaling Technology # Ac-K-103), RyR-total (Abcam #2827), CamKII-total (Santa Cruz #sc-9035, Dallas, TX, USA), NCX (Abcam #177952), and Cav1.2 (Alomone Labs #ACC-003, Jerusalem, Israel). For secondary antibodies, HRP-conjugated ECL-Anti mouse IgG (GE Healthcare #NA931, Marlborough, MA, USA) and ECL-Anti Rabbit IgG (GE Healthcare #NA934) were used. Blots were imaged with the ChemiDoc Touch (BioRad) and band intensity was quantified using Image Lab Software 6.0.1 (BioRad).

### 2.10. Statistical Analysis

Data management and statistical analyses were performed using Graph Pad PRISM 7.05 (La Jolla, CA, USA). All data are expressed as the mean ± the standard error. Comparisons of two independent groups were performed using a two-sided Student’s t-test for unpaired samples or Mann–Whitney test in the case where data were not normally distributed. Normality distribution was assessed by using Shapiro–Wilk normality test. When three or more groups were included, one-way ANOVA was performed. For the functional measurements in human trabeculae, a repeated measure two-way ANOVA was performed. If a significant interaction was identified, the Tukey post-hoc test for multiple comparisons was used. Two-sided testing was used for all statistical tests. A *p*-value of ≤0.05 was used to determine significance for all statistical tests. The data that supports the images and plots within this paper, as well as other findings from this study, are available from the corresponding author upon reasonable request.

## 3. Results

### 3.1. SAHA Improves Cardiomyocyte Contractility and Calcium Handling

**Figure 1:**
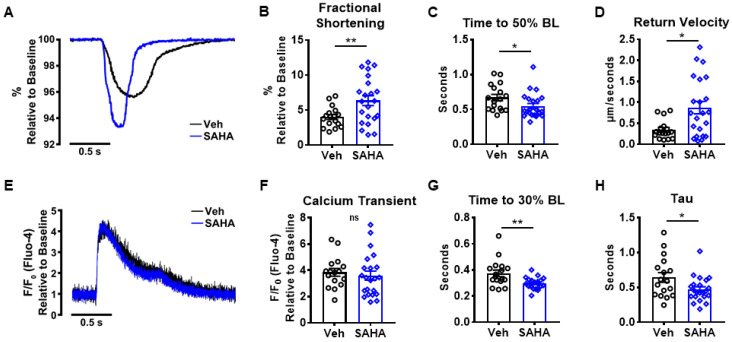
Effect of SAHA treatment on AFVM function. AFVM were isolated and incubated with SAHA or vehicle (2.5 μM) for 90 min, then incubated with Fluo-4am and Pluronic™ F-127 before functional measurements were acquired. (**A**) Representative contraction of SAHA treated vs. vehicle AFVM, which had an improvement in (**B**) fractional shortening, (**C**) Time to 50% baseline of contraction and (**D**) return velocity. (**E**) Representative calcium transient showing no difference between SAHA and vehicle-treated AFVM (**F**) peak calcium transient, but there was a decrease in (**G**) time to 30% baseline and (**H**) tau; n = 17–22 myocytes/parameter from 4 felines. Fractional shortening and tau were analyzed using two-sided Student’s *t*-test. Time to 50% BL, return velocity, calcium transient, and time to 30% BL were analyzed using Mann–Whitney test. NS stands for not significant. * *p* < 0.05, ** *p* < 0.01. Data shown are means ± SEM.

We assessed the acute effects of SAHA treatment on cardiomyocyte function using adult feline ventricular myocytes (AFVM) isolated from healthy males. Freshly isolated cells were treated with 2.5 µM SAHA or vehicle (Dimethyl sulfoxide (DMSO)) for 90 min and then paced using field stimulation (0.5 Hz). Once cells reached a steady state, contractions and calcium transients were recorded. Representative traces for the contraction and calcium transient are shown in Figure 1A,E. There was a significant increase in contraction magnitude assessed by fractional shortening in SAHA vs. vehicle cells (veh: 6.36 ± 0.71 vs. SAHA: 4.00 ± 0.36; *p* = 0.0099), in addition to faster relaxation kinetics (time to 50% baseline) (0.67 s ± 0.05 vs. 0.55 s ± 0.04; *p* = 0.0142) and return velocity (0.34 µm/s ± 0.05 vs. 0.87 µm/s ± 0.15; *p* = 0.0250) (Figure 1B–D). While there was no difference in peak calcium transients between groups (Figure 1F), there was a significant shortening of the recovery phase of calcium transients, reflected by the time to 30% of fluorescence signal decay (Time to BL 30%) in SAHA vs. veh-treated cells (0.37 s ± 0.02 vs. 0.30 s ± 0.01; *p* = 0.0044) (Figure 1G). The time to 30% return to BL was selected because mammals with long action potential duration have two phases of calcium reuptake with the first phase being primarily mediated by sarcoendoplasmic reticulum calcium transport ATPase (SERCA), while the second phase includes a contribution of the sodium–calcium exchanger (NCX), which is activated with repolarization [24]. Choosing 30% to BL is a conservative approach to capture primarily the first phase where SERCA is active. Tau, which is the time constant of the rate of cytosolic Ca^2+^ removal, was also significantly improved with SAHA vs. vehicle (0.64 s ± 0.07 vs. 0.47 s ± 0.04; *p* = 0.0276) (Figure 1H). Taken together, SAHA increased contraction magnitude and relaxation velocity and accelerated cytosolic Ca^2+^ removal without altering the peak calcium transients.

### 3.2. SAHA Treatment Increases Myofilament Calcium Sensitivity

**Figure 2:**
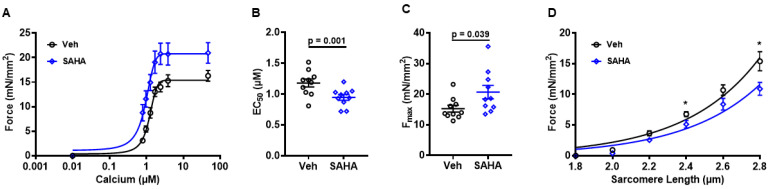
Effect of SAHA treatment on AFVM myofilament function. Skinned myocytes were isolated from AFVM to assess myofilament function. The (**A**) average force-calcium curves for SAHA and vehicle-treated skinned myocytes demonstrate a clear left shift with SAHA treatment. There was a decrease in the (**B**) EC_50_ with SAHA treatment, indicating an increase in myofilament calcium sensitivity and an increase in (**C**) maximal calcium-activated force (F_max_); n = 10 myocytes from 3 felines for each group. There was a decrease in (**D**) passive stiffness at increasing sarcomere lengths. n = 8 vehicle treated myocytes from 3 felines, n = 10 SAHA-treated myocytes from 3 felines. All analysis was performed using a two-sided Student’s *t*-test. * *p* < 0.05 Data shown are means ± SEM.

To assess the acute direct effect of SAHA on the sarcomere and differentiate myofilament from calcium cycling [25] effects, AFVM treated with 2.5 µM SAHA or vehicle for 90 min underwent a skinned myocyte isolation procedure. The membrane is exposed to triton detergent to chemically permeabilize all membranous structures including the nucleus, sarcolemma, sarcoplasmic reticulum, mitochondria, and other subcellular organelles, leaving only intact myofilaments [26]. This allows for direct assessment of myofilament function. Figure 2A shows average force–calcium relationship for SAHA and vehicle-treated skinned myocytes. There was a significant leftward shift with SAHA treatment. EC_50_ is the calcium concentration at which force is 50% of the maximum and representative of myofilament calcium sensitivity [23]. SAHA significantly reduced EC_50_ compared to veh (1.18 ± 0.07 µM vs. 0.95 ± 0.05 µM; *p* = 0.0092), indicating an increase in calcium sensitivity (Figure 2B). Maximal calcium-activated force (F_max_) was also significantly increased with SAHA treatment (15.24 ± 1.11 mN/mm^2^ vs. 20.68 ± 2.19 mN/mm^2^; *p* = 0.0397) (Figure 2C). Furthermore, there was a significant decrease in passive stiffness at increasing sarcomere lengths (Figure 2D). These data indicate that SAHA treatment significantly altered several indices of myofilament function, including calcium sensitivity, F_max_, and passive stiffness.

### 3.3. SAHA Treatment Alters Myosin Regulatory Light Chain Acetylation

**Figure 3:**
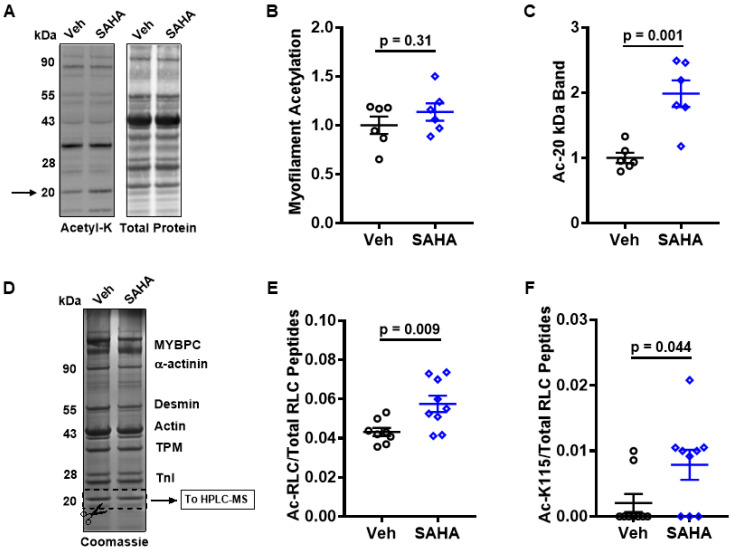
Effect of SAHA on myofilament acetylation. The myofilament acetylation was assessed in acutely treated AFVM (**A**–**F**). Western Blot analysis revealed that (**A**,**B**) total myofilament acetylation was not increased with SAHA treatment but there was significantly more (**C**) acetylation at a 20 kDa band (indicated with arrow in (**A**)); n = 6 felines per group. (**D**) Coomassie staining was performed, and the band was cut out for mass spectrometry analysis, which revealed that this 20 kDa band with (**E**) increased acetylation is the myosin regulatory light chain with one (**F**) specific residue (lysine 115) driving the increase in acetylation; n = 3 felines per group, 3 technical replicates per sample. All analysis was performed using a two-sided Student’s *t*-test. Data shown are means ± SEM

Next, we aimed to assess the underlying mechanisms of SAHA-related effects and identify substrates for acetylation/deacetylation that influence cardiac function. As an HDAC inhibitor, SAHA blocks the removal of lysine acetyl groups. Based on the improvement in AFVM myofilament function with SAHA treatment, we hypothesized that myofilament acetylation would be increased as well. There was no significant difference in global AFVM myofilament acetylation between groups treated with vehicle or SAHA (Figure 3A,B). However, there was one distinct band at approximately 20 kDa that displayed significantly increased acetylation (1.00 ± 0.08 vs. 1.99 ± 0.20; *p* = 0.0011) (Figure 3C). To confirm the identity of this 20 kDa band, it was run on an SDS-PAGE/Coomassie gel, excised, and analyzed by mass spectrometry (MS) (Figure 3D). The band contained primarily myosin regulatory light chain (RLC, MYL2, MLC-2), which was significantly acetylated with SAHA treatment (0.04 ± 0.002 vs. 0.06 ± 0.004; *p* = 0.0098) (Figure 3E). One specific RLC site, lysine 115 (K115), had a significant increase in acetylation (0.002 ± 0.001 vs. 0.01 ± 0.002; *p* = 0.0444) (Figure 3F).

### 3.4. SAHA Treatment Improves Developed Force and Diastolic Tension in Non-Failing Human Ventricular Myocardium

**Figure 4:**
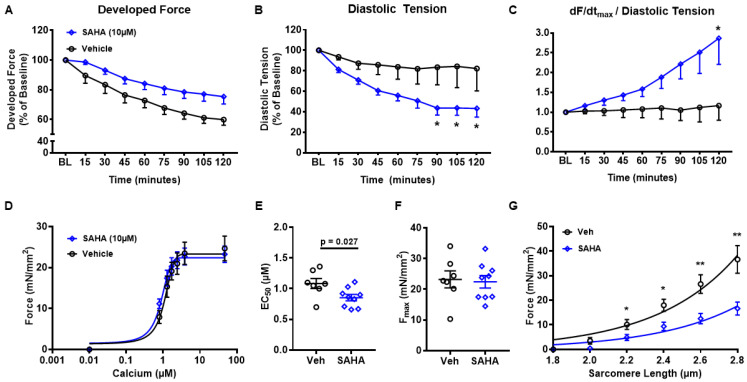
Effect of acute SAHA treatment on human trabeculae. Trabeculae were isolated from biopsies of non-failing human LV. There was an increase in (**A**) developed force and decreased (**B**) diastolic tension with SAHA treatment, with an increase in (**C**) dF/dt_max_/diastolic tension; n = 5–13 trabeculae per parameter from 7 patients. Myofilaments were isolated from human LV trabeculae. There was a slight left shift in the (**D**) average force-calcium curve with SAHA treatment. SAHA-treated samples had a reduction in (**E**) EC_50_, indicating an increase in myofilament calcium sensitivity but no difference in (**F**) F_max_; n = 7 control myocytes from 3 patients, 9 SAHA from 3 patients. SAHA-treated samples had a decrease in (**G**) passive stiffness at increasing sarcomere lengths; n = 7 myocytes from 3 patients in each group. For trabeculae functional experiments (**A**–**C**), repeated measure two-way ANOVA was performed. For the myofilament functional experiments (D-G), analysis was performed using a two-sided Student’s *t*-test. * *p* < 0.05, ** *p* < 0.01. Data shown are means ± SEM.

To validate the findings from AFVMs using a more translational approach, we repeated several experiments using left ventricle trabeculae isolated from non-failing human hearts. Trabeculae allow for the assessment of multicellular function in human intact myocardium [20,27,28,29,30,31]. For this study, trabeculae were used to assess the direct acute effects of SAHA. Ventricular trabeculae were isolated from non-failing human donor hearts, which were not suitable for transplantation (Table S1). SAHA-treated trabeculae had a less pronounced decrease in developed force over time (natural run down) (Figure 4A) with a similar systolic peak force (Figure S2A), although diastolic tension (Figure 4B) was significantly decreased. These findings are in line with the decreased passive stiffness we observed in vivo [5] and ex vivo. Furthermore, the maximum rate of force rise (dF/dt_max_) normalized to diastolic tension was increased by SAHA treatment (Figure 4C). The maximum rate of force decay (dF/dt_min_) was not different between groups (Figure S2B). These findings suggest increased contractility and decreased diastolic tension with SAHA treatment.

### 3.5. SAHA Treatment Enhances Myofilament Calcium Sensitivity in Non-Failing Human Ventricular Myocardium

Myofilaments were then isolated from the human trabeculae samples. Figure 4D shows the average force-calcium curves for SAHA and vehicle-treated samples. Just as in the SAHA-treated AFVM skinned myocytes, there was a significant decrease in EC_50_ (1.08 ± 0.08 vs. 0.85 ± 0.05; *p* = 0.0266) (Figure 4E) with no change between groups for F_max_ (Figure 4F). Consistent with our findings in AFVM, there was a significant decrease in passive stiffness at increasing sarcomere lengths with SAHA treatment (Figure 4G). Thus, SAHA treatment increases calcium sensitivity in human myocardium and at the same time reduces passive stiffness.

**Figure 5:**
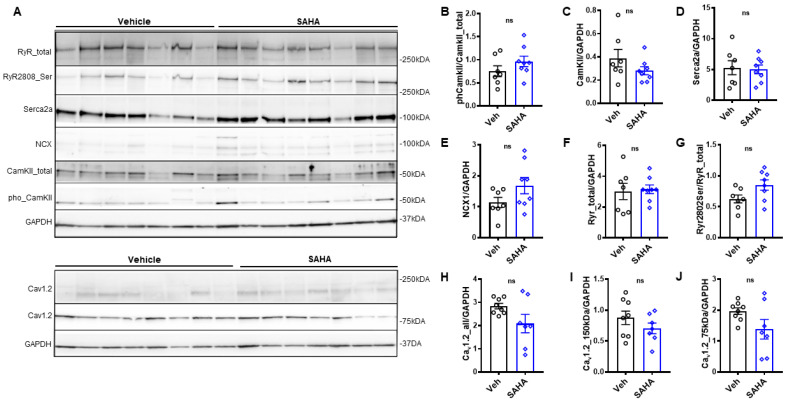
Effect of acute SAHA treatment on abundance of calcium handling proteins. Western Blots (**A**) of right atrium trabeculae after being treated with vehicle or SAHA. There was no change in protein abundance with SAHA treatment for (**B**) phosphorylated calcium/calmodulin-dependent protein kinase (pCaMKII T286), (**C**) CaMKII, (**D**) sarcoendoplasmic reticulum calcium transport ATPase (SERCA), (**E**) sodium–calcium exchanger 1 (NCX1), (**F**) ryanodine receptor (Ryr), (**G**) Ryr serine 2808, (**H**) Cav1.2, (**I**) Cav1.2 150 kDa, and (**J**) Cav1.2 75 kDa; n = 7–8 samples per protein from 8 patients. All analysis was performed using a two-sided Student’s *t*-test. NS stands for not significant. Data shown are means ± SEM.

We assessed the protein abundance of key cardiac calcium-handling proteins in human myocardium to assess if they are involved in SAHA-mediated effects. There was no significant difference in protein abundance of phosphorylated calcium/calmodulin-dependent protein kinase (pCaMKII T286), CaMKII, sarcoendoplasmic reticulum calcium transport ATPase (SERCA), sodium-calcium exchanger 1 (NCX1), ryanodine receptor (Ryr), Ryr serine 2808, Cav1.2, Cav1.2 150 kDa, and Cav1.2 75 kDa. These findings suggest that improvemenst in cardiac function with SAHA treatment are not due to changes in the abundance or activity levels of these proteins (Figure 5A–J).

### 3.6. Isoform Selective HDAC Inhibition Increases Developed Force in Dose Dependent Manner

**Figure 6:**
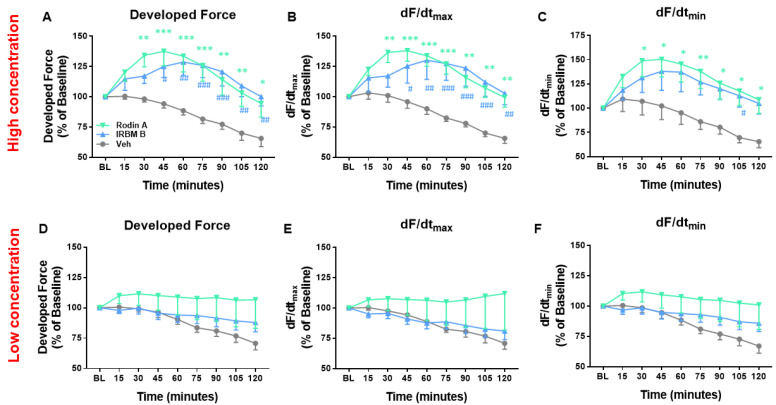
Effect of isoform selective HDAC inhibition on non-failing human trabeculae. Trabeculae were isolated from right atrial appendages (RAA) and treated with Rodin-A, IRBM-D, or vehicle at high or low concentrations. (**A**) Developed force, (**B**) dF/dt_max_, and (**C**) dF/dt_min_ were all increased with high concentrations of Rodin-A and IRBM-D compared to vehicle treatment. There was no significant difference (**D**) developed force, (**E**) dF/dt_max_, and (**F**) dF/dt_min_ in the lower concentration-treated groups compared to vehicle; high concentration: Rodin-A, n = 8 trabeculae; IRBM-D, n = 9 trabeculae; low concentration: Rodin-A, n = 7 trabeculae; IRBM-D, n = 13 trabeculae; vehicle, n = 8 trabeculae. RAA trabeculae were isolated from 30 patients. All analysis was performed using repeated measure two-way ANOVA. * *p* < 0.05, ** *p* < 0.01, *** *p* < 0.001 Rodin-A vs. vehicle, # *p* < 0.05, ## *p* < 0.01, ### *p* < 0.001 IRBM-D vs. vehicle. Data shown are means ± SEM.

To harness the potential of HDACi to treat cardiovascular diseases, safer compounds may be required to enable long-term treatment. Therefore, more selective inhibitors of HDACs were explored to assess whether improved selectivity could maintain efficacy and minimize the dose-limiting toxicities, but with limited success so far [21]. Italfarmaco has designed new HDACi core scaffolds that maintain the inhibitory activity toward HDAC1 and HDAC2 and optimize the structure−activity relationship (SAR) for hematological safety in patients. In order to determine if this improvement in myocardial function is conserved with a more targeted approach, we evaluated the effect of these isoform selective HDAC inhibitors on non-failing human atrial trabeculae. Two class I-selective HDAC inhibitors, Rodin-A (inhibits HDAC 1 + 2) and IRBM-D (inhibits HDAC 1 + 2 + 3) were tested at high and low concentrations vs. vehicle (DMSO). The higher concentrations of Rodin-A and IRBM-D both caused a significant acute increase in developed force vs. the vehicle-treated group (Rodin-A: 94.5% ± 11.3%, n = 8; IRBM-D: 100.2% ± 7.7%, n = 7; veh: 70.7% ± 5.4%, n = 8; *p* < 0.05) (Figure 6A). There was also an increase in dF/dt_max_ and dF/dt_min_ with the higher concentration treatment (Figure 6B,C). This effect appears to be dose dependent as lower concentrations of Rodin-A and IRBM-D have blunted effects on developed force and kinetics (Figure 6D–F). This data provides proof of concept for the potential effectiveness of isoform selective HDAC inhibitors in human myocardium.

## 4. Discussion

We previously [5] described the effects of SAHA in a large animal model of slow progressive pressure overload, recapitulating features of human HFpEF. We provided evidence supporting several potential mechanisms but there were many unanswered questions regarding how SAHA regulates cardiac function. This study was designed to develop a deeper understanding of how SAHA modulates function at the level of the cardiomyocyte and its contractile components. Furthermore, this study is the first to describe the effects of SAHA on human ventricular myocardium and extend these findings by performing parallel experiments using tissue derived from a large mammal with comparable physiological features. Using a systematic approach, we assessed the effect of SAHA on global cardiomyocyte function, myofilament function, and protein acetylation. Our study identified significant changes upon SAHA treatment in three cardiac properties—(1) increased contractility; (2) accelerated relaxation, and (3) decreased passive tension, suggestive of multiple sites of action on a cellular/molecular level.

### 4.1. SAHA Improves Contractility in Feline and Human Myocardium via Increasing Myofilament Calcium Sensitivity

In vivo SAHA treatment increased speckle-tracking-based global radial strain in our feline model [5]. In vitro data from the current study confirm these findings. SAHA increased fractional shortening in AFVMs and developed force in human non-failing myocardium. SAHA also induced a significant increase in the maximal rate of force rise (dF/dt_max_) when normalized to diastolic tension. These findings suggest that SAHA treatment exerts positive inotropic effects, as the trabeculae generated similar peak systolic forces at lower diastolic tension (preload) compared to the vehicle-treated group [32]. In HFpEF patients, limited systolic reserve also affects diastolic function because recoil and suction forces during early diastole are attenuated [33], thus, improved contractility by SAHA may indirectly improve diastolic function. It is important to note that the increase in contractility is not mediated via an increase in calcium transients, which is an unfavorable mode of action in patients with heart failure. Several clinical trials have reported increased mortality and progression of heart failure among patients treated with inotropes, which alter intracellular calcium concentrations due to increased myocardial oxygen demand and arrhythmias. Emerging therapies to improve cardiac function via optimizing metabolism (mitotropes; e.g., SGLT2-inhibitors) or increasing sarcomere function (myotropes; e.g., omecamtiv mecarbil) may provide useful alternatives in the future [34]. In this regard, SAHA might be another promising candidate, since inotropy is mediated via calcium-independent mechanisms. In skinned myocyte experiments, we showed that ex-vivo acute treatment of AFVM and human myofilaments resulted in increased calcium sensitivity with SAHA treatment, reflected by a leftward shift of the force-calcium curve. Myofilament calcium sensitivity reflects the contractile response of the myofilaments to a given calcium concentration [35]. Troponin I (TnI) phosphorylation on multiple residues via protein kinase A contributes significantly to calcium-sensitive force production and myofilament relaxation. A single phosphorylation of TnI at serine 23 and 24 accelerates relaxation, but decreases contractility, thus enhancement in myocardial calcium sensitivity may slow down relaxation [36,37]. In our study we found a hastened relaxation and ruled out the contribution of classic key cardiac calcium handling proteins, suggesting alternative and several mechanisms underlying the effects of SAHA.

### 4.2. SAHA Increases Relaxation by Accelerating Cytosolic Calcium Removal

We previously described that in vivo SAHA treatment shortened the phase of linear relaxation duration on a myofibrillar level, which correlated with invasively measured indices of diastolic function (tau, LVEDP) [5]. While there is a growing body of evidence describing the impact of HDAC inhibition on the cardiovascular system, there is little evidence describing the effects of SAHA on cardiomyocyte function. The studies currently available in the literature describe the effect on cardiomyocytes isolated from rodents [13,14]. Meraviglia et al. reported increased SERCA2 acetylation and activity after SAHA treatment in cardiomyocytes isolated from healthy adult rats [13]. This study found no change in the amplitude or time to peak calcium transient between control and SAHA treated groups, in line with our findings. Tau, used as a surrogate for cytosolic calcium clearing, which was improved in the SAHA-treated cells, as was the time to 10%, 50%, and 90% decay in the fluorescence signal. We also found a shortening in tau and time to 30% BL, representing accelerated calcium reuptake. Meraviglia et al. also reported increased SERCA ATPase activity with SAHA treatment in microsomes isolated from both rat cardiomyocytes and HL-1 cells (AT-1 mouse atrial cardiomyocyte tumor lineage), which would be an explanation for increased rate of calcium removal [13]. However, they did not report differences in the contractility of the cell, with no change in fractional shorting or maximal rate of shortening but did note an increase in the maximal rate of re-lengthening. In our study, we saw a significant increase in fractional shortening with no difference in peak calcium in SAHA vs. veh-treated AFVMs, which is suggestive of increased myofilament calcium sensitivity. Bocchi et al. [14] assessed the effects of SAHA on cell contractility and calcium dynamics in unloaded ventricular myocytes isolated from the heart of control and diabetic rats. Although fractional shortening was unaltered, the maximal rate of shortening (−dL/dt_max_) and time to peak calcium transient (TTP) were improved by SAHA. A potential explanation for the differences in results between this study and previous reports are differences in physiological properties between a rodent and a larger mammal, including prolonged action potential duration and myosin heavy chain composition (αMHC vs. βMHC) [15,16]. The physiological properties of felines more closely mimic those of humans, making the model highly valuable for translational research. We provide robust evidence of increased relaxation kinetics and cytosolic calcium removal with SAHA treatment. These effects together with improved myofibrillar relaxation (previously shown by our group) could explain the net improvements in in vivo diastolic function (reduction in LVEDP) seen in our model [5] despite an increase in myofilament calcium sensitivity.

The most consistent finding throughout the different aspects of this study is that SAHA decreases passive stiffness. Initially, diastolic tension was significantly decreased in intact trabeculae. Myofilaments isolated from treated AFVM and human trabeculae had a significant decrease in passive stiffness with SAHA treatment. The mechanism driving this beneficial functional change is unclear. We speculate that post-translational modification of the microtubule and/or titin may play a role.

### 4.3. Myosin Regulatory Light Chain Acetylation Is Increased with SAHA Treatment

Based on the evidence that HDACs can mediate post-translational modifications and the observed changes in myofilament function with SAHA treatment, assessing the myofilament for changes in acetylation was the clear next step. There was a significant increase in acetylation of a band at around 20 kDa, which mass spectrometry analysis revealed to be myosin regulatory light chain (RLC). More specifically, lysine 115 (K115) was heavily acetylated with SAHA treatment. The RLC and essential light chain (ELC) are part of the neck region of the myosin motor that is an essential part of muscle contraction [38]. Both the RLC and ELC can alter muscle contraction by mediating changes in myosin motor activity, again highlighting their importance in the function of muscle [38]. While few studies have looked at acetylation of myosin or the associated light chains, two proteomic-based reports have described cardiac acetylation of different species. The guinea pig cardiac lysine acetylome had similar acetylation to the AFVM in this study but did not note K115 [39]. In another study, top-down mass spectrometry was performed in both human and swine atrial and ventricular tissue. Both human and swine atrial RLC were found to be N^α^-acetylated, but the significance of this finding was unclear and once again K115 was not noted [40]. Both studies described the proteome but did not report any functional findings to supplement the characterization. In this study, we narrowed our focus to the cardiomyocyte and myofilament to better understand how SAHA exerts its beneficial effects and provide evidence for a potential novel mechanism for pan-HDAC inhibition to directly impact cardiac function. Previous studies have reported co-localization of HDAC2 and myofibrils but did not report the substrate [41]. We provided evidence of a substrate via the acetylation of RLC K115. The current literature on cardiac acetylation is more descriptive and lacks functional analysis [39,40], but our study provides a functional assessment in addition to the proteomic data.

Since previous clinical trials using pan-HDAC inhibitors reported adverse effects such as leukocytopenia, thrombocytopenia, gastrointestinal symptoms, and QT interval prolongation [42], a more targeted approach may be needed to improve the safety profile of this class of drugs. Therefore, we assessed the functional effects of isoform-selective HDAC inhibitors, which are not commercially available. In this proof-of-concept study, we found a dose-dependent increase in developed force and twitch kinetics in human atrial tissue when inhibiting class 1 HDACs 1 + 2 using Rodin-A and HDACs 1 + 2 + 3 using IRBM-D. Future studies are warranted to delineate molecular and myofilament changes.

We report for the first time the effects of SAHA on human cardiac tissue and provide functional insights using live twitching human trabeculae. In the current study, we have provided evidence of a potential new mechanism for pan-HDAC inhibition improving cardiac function by improving myofilament calcium sensitivity. Importantly, we identified a potential substrate for hyperacetylation via SAHA that may be in direct control of cardiac contractility. The impact on relaxation is likely explained by (1) faster diastolic Ca^2+^ removal from the cytosol seen in isolated AFVMs; (2) improved myofibril relaxation [5] (recently reported by our group); and (3) improved contractility, which affects recoil and suction force during diastole (in vivo) [33]. We could not determine what underlying mechanisms are driving the improved passive tension, but it might be due to acetylation changes on myofilament proteins, titin, and/or microtubules as well.

Since the experiments described above were performed using a relatively acute period of HDAC inhibition treatment, the beneficial effects are likely due to post-translational modification by a direct effect of increased myofilament protein acetylation and not due to epigenetic effects (altering gene expression). It is important to point out, however, that we previously studied the effect of chronic SAHA treatment [5], which may lead to changes in gene expression and likely have broader cellular effects. Thus, we cannot rule out whether elevated RLC acetylation and myofilament calcium sensitivity is limited to the acute phase or if these are lasting changes. Future studies are needed to parse out the effects of acute and chronic SAHA administration.

These findings suggest that SAHA exerts multiple effects to culminate in improved cardiac function. The experiments performed were essential for laying the foundation for future studies that can have a more targeted focus on delineating mechanistic insights.

## 5. Limitations

Human HFpEF is a complex clinical syndrome and cannot be fully recapitulated in any animal model. However, the feline model used does capture several key clinical characteristics (i.e., elevated filing pressures, diastolic dysfunction, pulmonary hypertension, LV hypertrophy, LA dilation, and impaired function), making it a suitable platform for testing therapies. These animals are young (2 months of age at start of study) and have no metabolic comorbidities. Non-failing human cardiac tissue is rare and samples are available in limited quantities. This constrained the analyses that could be performed and did not allow for the development of an in-depth molecular component of the study using human cardiac tissue. Western blot analysis for abundance of key calcium handling proteins was performed using atrial tissue. While ventricular tissue would have been ideal, freshly harvested atrial tissue was available, and this allowed for us to assess the effect of SAHA compared to vehicle on live human myocardium. While we focused primarily on the myofilament, changes to titin or microtubules could also play a role in mediating the functional improvements observed. We did not perform any titin or microtubule-related experiments as they were beyond the scope of the study.

## Data Availability

Data is contained within the article or supplementary material.

## Supplementary Materials

The following supporting information can be downloaded at: https://www.mdpi.com/article/10.3390/pharmaceutics14071509/s1, Figure S1: Study Design; Figure S2: Effect of SAHA on trabeculae function; Table S1: Patient Characteristics.
