# Supplementary material for: HDAC Inhibition Regulates Cardiac Function by Increasing Myofilament Calcium Sensitivity and Decreasing Diastolic Tension
Source: Pharmaceutics. 2022 Jul 21;14(7):1509. doi: 10.3390/pharmaceutics14071509 (PMC9323146; doi:10.3390/pharmaceutics14071509)
Supplement: Supplementary file 1 [file pharmaceutics-14-01509-s001.zip › pharmaceutics-1798029-supplementary.pdf]

Supplemental Figures

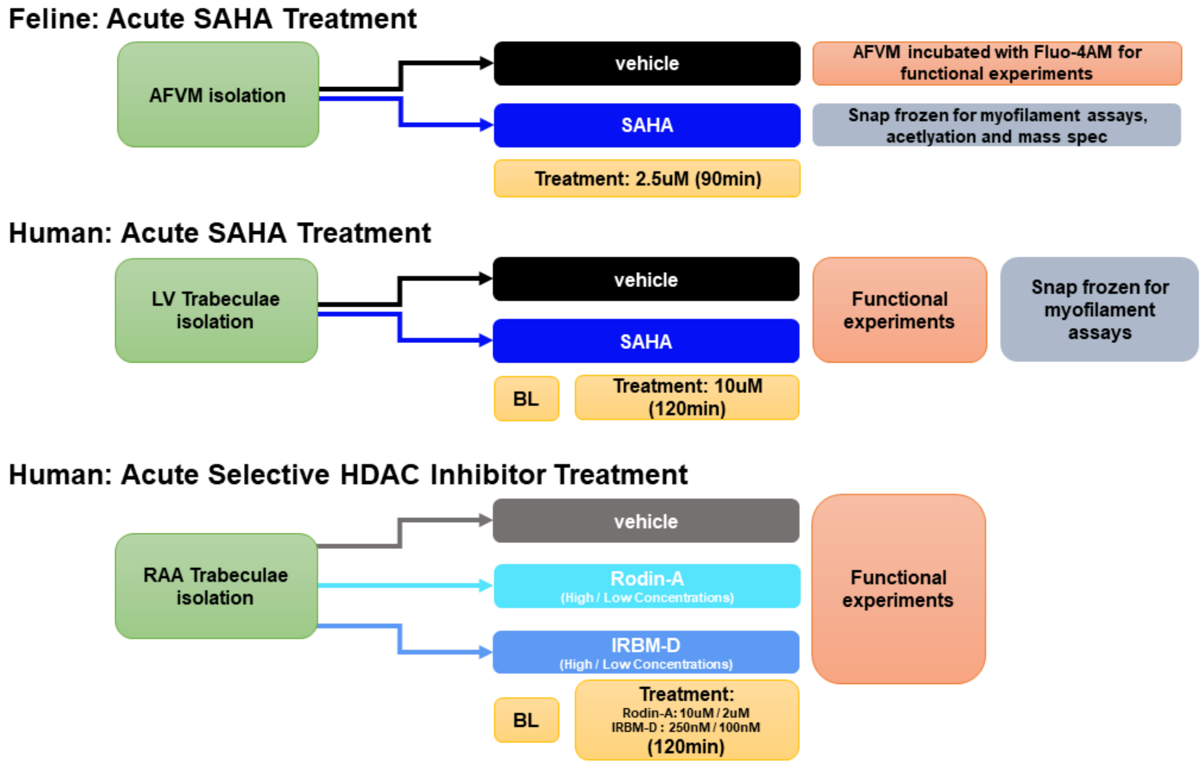

Figure S1. Study Design

Adult feline ventricular myocytes (AFVM) were isolated from domestic short hair felines (n=4, 6 months old) and treated with either vehicle or SAHA for 90 minutes. AFVM were then incubated with Fluo-4AM for functional experiments to assess contractility and calcium handling or snap frozen for myofilament assays, acetylation, and mass spectrometry. Trabeculae were isolated from ventricles of human non-failing donor hearts that were not suitable for transplantation or right atrial appendages (RAA) (LV: n=7 patients, RAA: n=30 patients). LV trabeculae were treated with vehicle or SAHA for 120 minutes for functional experiments before being snap frozen for myofilament assays. RAA trabeculae were treated with vehicle or a low/high dose of a selective HDAC inhibitor for 120 minutes for functional experiments.

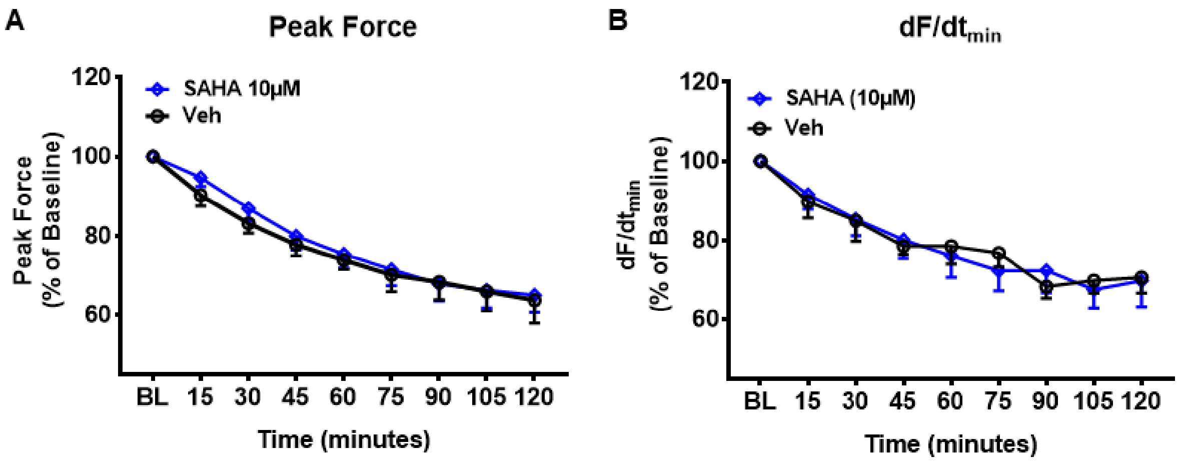

Figure S2. Effect of SAHA on trabeculae function
Trabeculae treated with SAHA generated similar (A) peak force and (B) dF/dt_{min} compared to vehicle treatment; n=5-13 trabeculae per parameter from 7 patients. repeated measure two-way ANOVA was performed. *p < 0.05, **p < 0.01, ***p < 0.001. Data shown are means ± SEM.

Supplemental Table

Table S1. Patient Characteristics

	Female	LV EF %	Age (years)	Body Mass Index (kg/ m²)
**LV trabeculae Non-failing donors (n=7)**	3	62 ± 5	60 ± 7	33 ± 11
Low concentration: **RAA trabeculae Non-failing donors (n=18)**	2	60 ± 6	65 ± 10	26.5 ± 4.8
High concentration: **RAA trabeculae Non-failing donors (n=12)**	2	60 ± 8	68 ± 9	29 ± 5
